# DDX49 is a novel biomarker and therapeutic target for lung cancer metastases

**DOI:** 10.1111/jcmm.14734

**Published:** 2019-11-20

**Authors:** Xiaojuan Lian, Debing Xiang, Chunfang Peng, Jiangyan Chen, Maojun Liao, Guiyin Sun, Zhimin Zhang

**Affiliations:** ^1^ Oncology Jiangjin District Central Hospital Chongqing China; ^2^ Cancer Center Daping Hospital Amry Medical University Chongqing China

**Keywords:** DDX49, lung cancer, lymph node metastases, predictor and therapeutic target

## Abstract

The identification of lymph node metastases is important for the diagnosis, treatment and prognosis of patients with lung cancer. We found DDX49 was associated with the lymph node metastases in lung cancer by the Akt/β‐catenin pathway. Transcriptome sequencing, bioinformatics analysis, quantitative RT‐PCR, cell transfection and the Cancer Genome Atlas (TCGA) data set were used to identify DDX49 responsible for lymph node metastases. A Kyoto Encyclopedia of Genes and Genomes (KEGG) pathway enrichment analysis was used to explore the possible molecular mechanism in experimental cell. The DDX49 gene was correlated significantly with lymph node metastases of lung cancer. The knockdown of DDX49 inhibited the cell proliferation and migration in PC‐9 and H460 cells. The mechanism research found downexpression of DDX49 decreased the Akt/β‐catenin pathway in lung cancer cell. In vivo experiments showed that DDX49 promoted the proliferation and metastases of lung cancer cells by increasing the Akt/β‐catenin pathway. These findings suggested that DDX49 may be useful as a novel biomarker of lymph node metastases and therapeutic target for lung cancer metastasis.

## 1 INTRODUCTION

The highest occurrence and fatality rate was lung cancer in the world. The accurate diagnosis, especially the diagnosis of distant metastases, is one of the important factors to improve the prognosis of lung cancer therapy. The first diagnosis of lung cancer is often with distant metastases, especially lymph node metastases, so patients have poor 5‐year survival rates. Lymph node metastases have a higher incidence rate and are not only one of the most important factors determining the cancer stage but also an important indicator of treatment options in lung cancer^1^. The current diagnosis for lymph node metastases of lung cancer has not a precise maker. Only less than 80% accuracy in detecting lymph node metastases is achieved by enhanced CT, which is the first choice of diagnostic methods^2^. The approximately 20% of lymph node metastases remain undetected by the routinely methods. Patients with undetected T3 metastases will not benefit from radical surgical resection and may experience postoperative recurrence^3^. In addition, this is not a precise target of lymph node metastases. Therefore, it is essential to look for a new marker for individualized treatment options to accurately consider the stage of the cancer to decrease adverse events in patients without necessary treatment. In our research, we found that DDX49 may be useful as a novel biomarker of lymph node metastases and therapeutic target for lung cancer metastasis.

## 2 METHODS AND MATERIALS

### 2.1 TCGA data set

A preprocessed expression matrix of gene‐level RSEM values from 188 cases of lung cancer originally documented in TCGA data sets (Table S1 clinical characteristics of lung cancer patients) (https://tcga.xenahubs.net/download/TCGA.LUNG.sampleMap/AgilentG4502A_07_3.gz). Clinical information of this TCGA cohort was also obtained from UCSC (https://tcga.xenahubs.net/download/TCGA.LUNG.sampleMap/LUNG_clinicalMatrix.g).

### 2.2 Western blot analysis

Equal amounts of proteins were separated on SDS‐polyacrylamide gels, transferred to PVDF membranes and then blocked. Next, membranes were incubated with primary antibodies, followed incubation with horseradish peroxidase‐conjugated secondary antibodies. Finally, blots were imaged using chemiluminescent staining reagents.

### 2.3 Scratch assay and Transwell assay

The cell‐culture inserts were coated with 5 µL pure Matrigel (Sigma) and placed in a 24‐well plate. Cells were detached, and single‐cell suspensions were placed into the upper chamber. After 18 hours, the filters were fixed and stained with crystal violet. The invasive cells on the lower surface of the filters were examined by bright field microscopy. Cells were plated in 6‐well culture plates and incubated. Cell monolayers were then scratched and washed. The wounded areas were imaged using an Olympus microscope and marked. The same areas were imaged again to observe the wound gap.

### 2.4 Patients

Ten specimens that used transcriptome sequencing and bioinformatics analysis were obtained from surgically resected primary tissue in patients with lung cancer treated at Daping Hospital of the Army Medical University (China) and were frozen and kept in liquid nitrogen (Table S2 clinical characteristics of lung cancer patients).

### 2.5 Statistical analysis

Fifty‐eight significantly differentially expressed genes were analysed to identify candidates potentially predictive for lymph node metastasis using univariate logistic regression. One gene was found associated with lymph node metastasis with crude *P* value <.05. All other statistical analyses were performed using SPSS 17.0 (IBM SPSS). All tests were bilateral, and *P* < .05 was considered statistically significant.

## 3 RESULTS

### 3.1 DDX49 gene was associated with lymph node metastases of lung cancer

**Figure 1:**
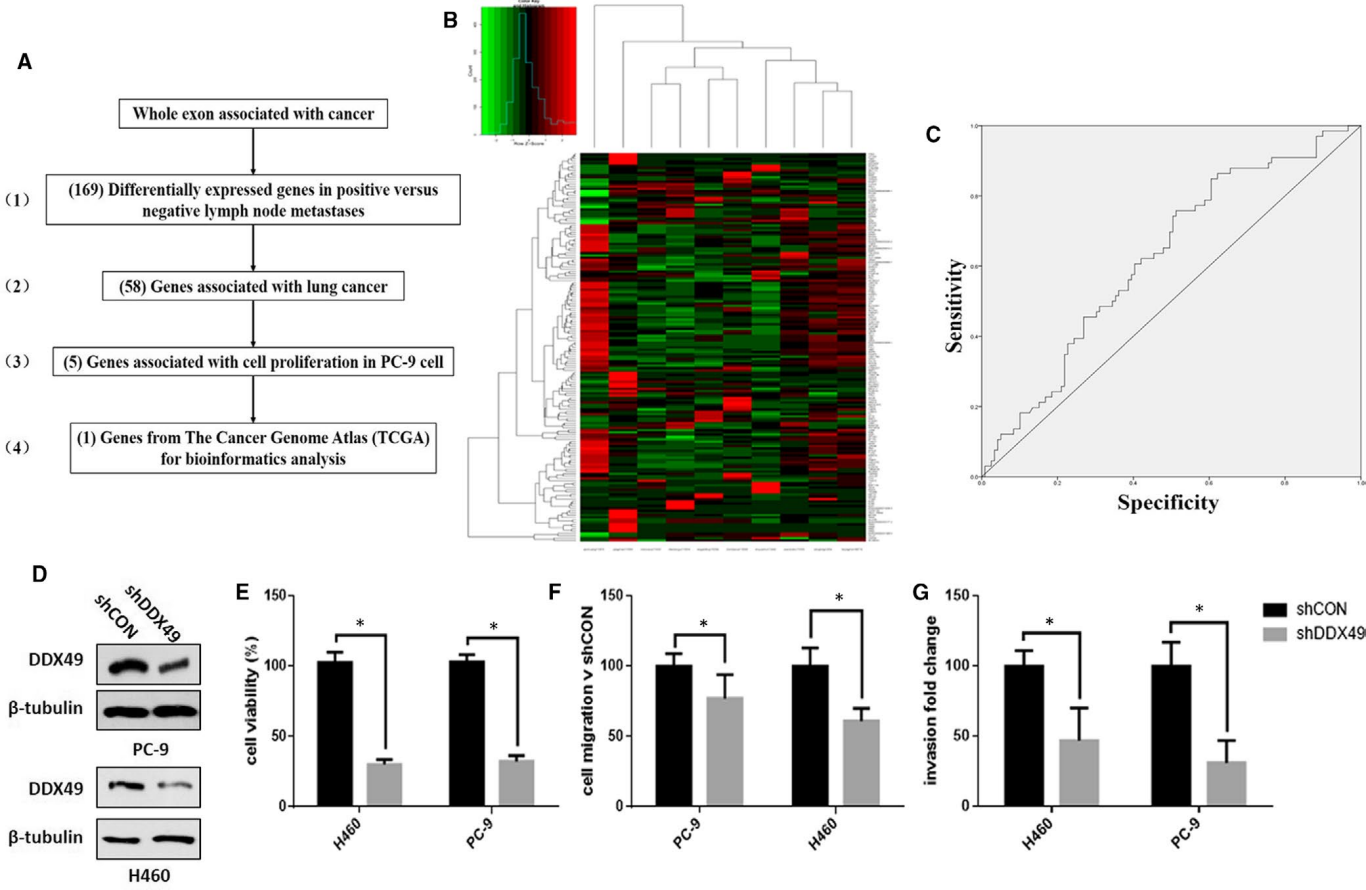
DDX49 was key gene for lung cancer proliferation and metastases. Overall strategy for the identification of 58 lymph node metastases‐associated genes in human lung cancer. (A) Genes associated with lymph node metastases of lung cancer were defined as the overlapping genes between three distinct conditions, namely genes differentially expressed in 5 paired positive and negative lymph node metastases from transcriptome sequencing, genes expressed in lung cancer from RT‐PCR, genes expression associated with cell proliferation and gene associated with lymph node metastases from bioinformatics analysis of TCGA data from the transcriptome sequencing results from 188 samples. We used a false discovery rate <0.05 and a |FC|> 1 to select differentially expressed genes. FC, fold change; HR, hazard ratio. (B) The 169 genes differentially expressed in 5 paired positive and negative lymph node metastases from transcriptome sequencing; (C) the ROCs of the DDX49 obtained using logistic regression with risk factors. The AUC, sensitivity and specificity of DDX49 for prediction of lymph node metastases were 62.4%, 75.8% and 51.3%, respectively. Effects of DDX49 on lung cancer cell growth, migration and invasion in vitro. Western blot analysis showed DDX49 down‐regulation in PC‐9 cells and H460 cells. Down‐regulation of DDX49 (D) in lung cancer cells significantly decreased cell proliferation (E), migration (F) and invasion (G) abilities compared with that of control cells. Data represent the average of three independent experiments (mean ± SD). *P < .005

**Figure 2:**
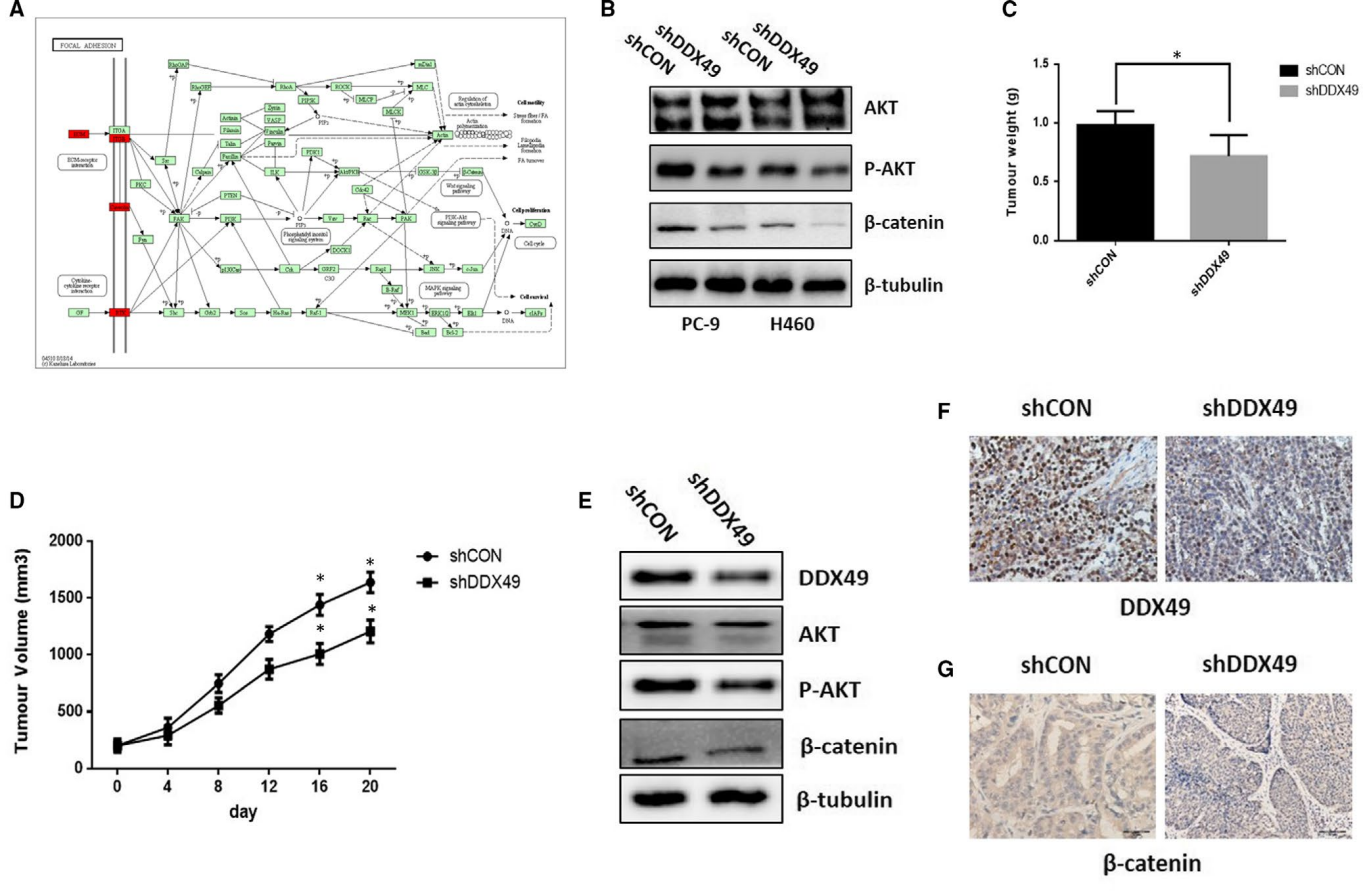
Effects of DDX49 on lung cancer cell growth and metastases in vitro and vivo. (A) Focal adhesion pathway in KEGG; the biological relationships that correspond to the core edges are annotated in red. (B) Western blot showed that down‐regulation of DDX49 decreased the levels of p‐Akt and β‐catenin protein. Nude mice were injected with H460 cells stably transfected with a plasmid containing shDDX49. Xenograft models were divided into two groups (10 mice/group) and treated for 14 days. Tumour weight was measured on day 14 (C). Tumour volume was measured using a calliper (D). Tumour tissues isolated from xenografts were subjected to Western blot analysis (E) and immunohistochemistry analysis (F and G). The data are shown as the mean ± standard error, n = 10. * *P* < .01 versus the vehicle group

To research the molecular nature of lymph node metastases in lung cancer, a sequential approach was used to select genes associated with lymph node metastases (Figure 1A). We assessed the gene expression profiles of 10 lymph nodes representing the same spectrum of lung cancer obtained from different individuals (five patients with positive lymph node metastases and five patients with negative lymph node metastases). This comparison identified 169 genes with differential expression patterns that best distinguished lymph node metastases from non‐lymph node metastases (1). The results are displayed as a heat map (Figure 1B); (2) 58 genes were associated with lung cancer; (3) 5 genes were associated with cell proliferation in lung cancer cell; and (4) DDX49 gene was further identified in TCGA data of 188 sample transcriptome sequencing results (Figure 1C), with a crude *P* value of <.05. To identify the predictive value of DDX49 for lymph node metastases of lung cancer, we analysed the exon sequencing data of 188 patients with lung cancer from TCGA and obtained 5 genes that were associated with lymph node metastases and cell proliferation, with a crude *P* value of <.05, through multivariate logistic regression. The prediction rate (area under the curve (AUC)), sensitivity (true positives) and specificity (false positives) of the risk score for the prediction of lymph node metastases were 82.0%, 84.8% and 65.0%, respectively (Figure 1C). The AUC, sensitivity and specificity of DDX49 for prediction of lymph node metastases were 62.4%, 75.8% and 51.3%, respectively. Taken together, DDX49 was differentially expressed in positive versus negative lymph node metastases, expressed in lung cancer cell, and associated with the proliferation and lymph node metastases of lung cancer in the exon sequencing data of 188 patients from TCGA.

### 3.2 Effects of DDX49 on lung cancer cell growth and migration

Then, we investigated the role of DDX49 in cell proliferation, migration and invasion in lung cancer cells by a lentivirus‐knockdown assay. Down‐regulation of DDX49 in PC‐6 and H460 cells, as shown in Figure 1D, substantially decreased cell growth, migration and invasion (Figure 1E‐G).

Moreover, we applied immunoblotting to identify KEGG pathways associated with lymph node metastases of lung cancer, with a corrected *P* < .0001 and expression in more than half of the samples (Table S3). KEGG pathway enrichment analysis of all genes selected from all differentially expressed genes showed that the PI3K/Akt and Wnt/β‐catenin pathways may play key roles in the metastases of lung cancer (Figure 2A). We demonstrated that DDX49 down‐regulation decreased Akt and β‐catenin activation (Figure 2B). These findings indicated that decreases in the activation of Akt/β‐catenin signalling by DDX49 knockdown may play a key role in inhibiting cell proliferation and migration in vitro.

### 3.3 Down‐regulation of DDX49 inhibited tumour growth in vivo

We studied the impact of DDX49 on cell growth in lung cancer cells by injecting H460 cells containing either a control (shCON) or DDX49‐down‐regulation vector (shDDX49) into nude mice in vivo. We observed that down‐regulation of DDX49 significantly decreased tumour growth in vivo (Figure 2C,D). Consistent with observations in vitro, shDDX49 decreased DDX49 expression in vivo (Figure 2E,F), and DDX49 down‐regulation decreased Akt and β‐catenin activation (Figure 2E,G). These findings indicated that DDX49 down‐regulation decreases the activation of Akt/β‐catenin signalling and may play a key role in inhibiting cell proliferation and migration in vivo.

## 4 DISCUSSION

In the present study, we integrated transcriptome sequencing data of lymph node metastases in lung cancer and an independent sample from TCGA data to identify DDX49 gene and validated the biological function. Furthermore, we demonstrated, for the first time, that DDX49 promoted NSCLC (non‐small‐cell lung cancer) cell growth and migration by increasing the Akt/β‐catenin pathway. Taken together, these findings suggested that DDX49 may be useful as a novel biomarker of lymph node metastases and a therapeutic target in lung cancer.

Our selection of DDX49 gene in the microarray detection set and the patterns of gene expression found on microarray analysis were also validated by RT‐PCR. Our results were validated in one independent cohorts of 188 patients from TCGA data set. Thus, we believe that the DDX49 we obtained using the three factors is reliable.

The identification of DDX49 gene that can predict lymph node metastases in patients with lung cancer may reveal a new target of therapy for lung cancer with lymph node or distant metastases. DDX49 is one such uncharacterized RNA helicase^4^, which has been implicated in viral infections and breast cancers in high‐throughput screens, suggesting an important physiological role^5^. We found that down‐regulation of DDX49 in NSCLC cells significantly suppressed NSCLC cell growth and migration. Sharad Awasthi also proposed a similar conclusion: DDX49 displays a robust ATPase and RNA helicase activity and it is involved in the export of poly (A)^+^ mRNAs to cytoplasm, DDX49 affects cell proliferation and its aberrant expression might have oncogenic potential^6^. We found that these genes were more likely involved in the PI3K/Akt pathway, cell and metabolic pathways, and focal adhesion by KEGG pathway analysis. Furthermore, we found that DDX49 mediated by the Akt/β‐catenin pathway promoted NSCLC cell growth and metastases. Growing evidence has shown that the Akt/β‐catenin pathway plays a key role in lung cancer cells^7^, ^8^. Taken together, DDX49 plays an important role through the Akt/β‐catenin pathway in increasing cell growth and metastases.

In conclusion, DDX49 was a novel predictor for diagnosing lymph node metastases of lung cancer; thus, the marker can be used to improve or even replace enhanced CT for the diagnosis of lymph node metastases. Moreover, the inhibition of DDX49 can significantly inhibit cell proliferation and metastases by the Akt/β‐catenin pathway.

## CONFLICT OF INTEREST

None declared.

## AUTHORS’ CONTRIBUTIONS

ZM. Z. and XJ. L. designed the study. MJ. L. GY. S. DB. X. and CF. P. performed the experiments. MJ. L. GY. S. DB. X. CF. P. and CF. P. analysed the results. ZM. Z. and XJ. L. wrote the manuscript. All the authors approved the final version.

## Data Availability

All data generated or analysed during this study are included in this article.

## References

[1] Ettinger DS , Wood DE , Akerley W , et al. Non‐small cell lung cancer, version 6.2015. J Natl Compr Canc Netw. 2015;13(5):515‐524. 25964637 10.6004/jnccn.2015.0071

[2] Zhou H , Liu JK , Chen SX , et al. Lymphatic microvessel density combined with CT used in the diagnosis of mediastinal and hilar lymph node metastasis of non‐small cell lung cancer. Arch Med Res. 2012;43(2):132‐138. 22386563 10.1016/j.arcmed.2012.02.002

[3] Naruke T , Goya T , Tsuchiya R , Suemasu K . The importance of surgery to non‐small cell carcinoma of lung with mediastinal lymph node metastasis. Ann Thorac Surg. 1988;46(6):603‐610. 2848463 10.1016/s0003-4975(10)64717-0

[4] Cordin O , Banroques J , Tanner NK , Linder P . The DEAD‐box protein family of RNA helicases. Gene. 2006;367:17‐37. 16337753 10.1016/j.gene.2005.10.019

[5] Apostolou P , Toloudi M , Papasotiriou I . Identification of genes involved in breast cancer and breast cancer stem cells. Breast Cancer (Dove Med Press). 2015;7:183‐191. 26203276 10.2147/BCTT.S85202 PMC4507490

[6] Awasthi S , Verma M , Mahesh A , et al. DDX49 is an RNA helicase that affects translation by regulating mRNA export and the levels of pre‐ribosomal RNA. Nucleic Acids Res. 2018;46(12):6304‐6317. 29618122 10.1093/nar/gky231 PMC6158705

[7] Liang CH , Chiu SY , Hsu IL , et al. alpha‐Catulin drives metastasis by activating ILK and driving an alphavbeta3 integrin signaling axis. Cancer Res. 2013;73(1):428‐438. 23047866 10.1158/0008-5472.CAN-12-2095

[8] Zhao M , Xu P , Liu Z , et al. Dual roles of miR‐374a by modulated c‐Jun respectively targets CCND1‐inducing PI3K/AKT signal and PTEN‐suppressing Wnt/beta‐catenin signaling in non‐small‐cell lung cancer. Cell Death Dis. 2018;9(2):78. 29362431 10.1038/s41419-017-0103-7 PMC5833350

