# Supplementary material for: DDX49 is a novel biomarker and therapeutic target for lung cancer metastases
Source: J Cell Mol Med. 2019 Nov 20;24(1):1141–5. doi: 10.1111/jcmm.14734 (PMC6933356; doi:10.1111/jcmm.14734)
Supplement: Supplementary file 1 [file JCMM-24-1141-s001.docx]

#### Supplemental table

**Supplemental table 1.** Clinical characteristics of 188 lung cancer patients from the TCGA dataset

|  | | Count | Column N % |
| --- | --- | --- | --- |
| gender | Female | 58 | 33.3% |
|  | Male | 124 | 66.7% |
| Histology | Adenocarcinoma | 32 | 17.2% |
|  | Squmous and others | 154 | 82.8% |
| T | T1 | 42 | 22.6% |
|  | T2 | 119 | 64.0% |
|  | T3-T4 | 25 | 13.4% |
| N | N0 | 119 | 64.3% |
|  | N1-N3 | 66 | 35.7% |
| M | M0 | 176 | 96.7% |
|  | M1 | 6 | 3.3% |
| Stage | I-II | 143 | 77.7% |
|  | III-IV | 41 | 22.3% |

**Supplemental table 2**. Clinical characteristics of 10 lung cancer patients for the genome-wide exon sequencing of lymph node blocks

| patients | SEX | Age | ID | Smoking | Pathological | lymph node metastasis | EGFR gene mutation | TNM stage |
| --- | --- | --- | --- | --- | --- | --- | --- | --- |
| number |  |  |  |  |  |  |  |  |
| 1 | male | 58 | 744472 | NO | Adenocarcinoma | NO | L858R | IIA |
| 2 | male | 51 | 775032 | NO | Adenocarcinoma | NO | 19Del | IIB |
| 3 | male | 70 | 775772 | NO | Adenocarcinoma | NO | L858R | IIB |
| 4 | male | 73 | 770449 | NO | Adenocarcinoma | NO | L858R | IIB |
| 5 | male | 58 | 761502 | NO | Adenocarcinoma | NO | L858R | IIA |
| 6 | male | 64 | 734080 | NO | Adenocarcinoma | YES | L858R | IIA |
| 7 | male | 68 | 740786 | NO | Adenocarcinoma | YES | L858R | IIA |
| 8 | male | 66 | 270216 | NO | Adenocarcinoma | YES | L858R | IIB |
| 9 | male | 67 | 764199 | NO | Adenocarcinoma | YES | L858R | IIB |
| 10 | male | 68 | 760026 | NO | Adenocarcinoma | YES | 19Del | IIIA |

**Supplemental table 3.** KEGG pathway of DEGs with corrected P< 0.0001 and expression in more than half of the samples.

| **Term** | **Database** | **Sample number** | **Background number** | **P-Value** | **Corrected P-Value** |
| --- | --- | --- | --- | --- | --- |
| PI3K-Akt signaling pathway | KEGG PATHWAY | 8 | 347 | 3.45E-05 | 7.76E-04 |
| Metabolic pathways | KEGG PATHWAY | 7 | 1218 | 0.2036 | 2.91E-01 |
| Focal adhesion | KEGG PATHWAY | 7 | 207 | 9.26E-06 | 3.12E-04 |
| Complement and coagulation cascades | KEGG PATHWAY | 7 | 69 | 5.23E-09 | 7.06E-07 |
| Pathways in cancer | KEGG PATHWAY | 7 | 398 | 0.000529 | 5.49E-03 |
| Phagosome | KEGG PATHWAY | 5 | 155 | 0.000216 | 2.92E-03 |
| ECM-receptor interaction | KEGG PATHWAY | 5 | 87 | 1.39E-05 | 3.76E-04 |
| Cell adhesion molecules | KEGG PATHWAY | 5 | 145 | 0.000159 | 2.68E-03 |
| Tight junction | KEGG PATHWAY | 5 | 138 | 0.000126 | 2.43E-03 |
| Malaria | KEGG PATHWAY | 5 | 49 | 8.01E-07 | 5.41E-05 |

**Supplemental figure**


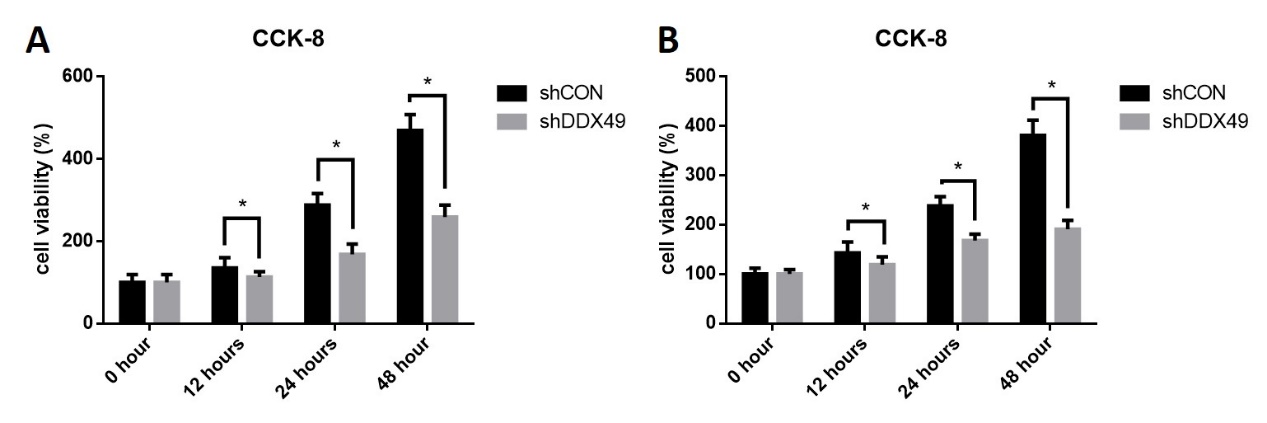


**The CCK-8 assay.** DDX49 knockdown plasmids (shDDX49) were transfected, PC-9-shCON/ PC-9-shDDX49 (A) and H460-shCON/ H460-shDDX49 (B) were seeded into 96-well plates for different time. Cell proliferation was determined by the CCK-8 method. Data represent the average of three independent experiments (mean ± SD). * P < 0.05, ** P < 0.01, vs. control.
